# Chronic Disease Self-Management: Behavioral Intervention Preferences Informed by the PEN-3 Cultural Model

**DOI:** 10.1093/geroni/igaa057.1547

**Published:** 2020-12-16

**Authors:** Chivon Mingo, Collins Airhihenbuwa

**Affiliations:** Georgia State University, Atlanta, Georgia, United States

## Abstract

Chronic Disease Self-Management Program (CDSMP) is an evidence-based program shown to improve health status, healthcare utilization, and health behaviors among individuals diagnosed with chronic conditions. Aging African Americans, a population at a greater risk of chronic disease diagnosis and burden, are underrepresented in the utilization of self-management behavioral programs. Previous research suggested that cultural distinctions (e.g., values, beliefs, preferences, experiences) impact the participation of racial/ethnic minorities in health-related research, health behavior outcomes, and healthcare utilization. Little is known about unique cultural influences on CDSMP utilization among this target group. To our knowledge, no research has applied a culture-specific theory to understand preferences or use of CDSMP among aging African Americans. Guided by the PEN-3 cultural model, this study examines preferences, barriers, and facilitators that may influence CDSMP utilization for the management of diagnosed chronic conditions. Using a qualitative research design, African Americans (N=50) from six Atlanta Metropolitan faith-based organizations participated in the 6-week CDSMP and one of six focus groups. Participants were largely female (70%) experiencing multiple chronic conditions (M=2) with an average age of 70. Focus groups were transcribed, and a thematic analysis was applied to identify emergent themes. Participants preferred programs that included family, community liaisons, and relevant advertisement material suggesting the importance of cultural identity. Accessing CDSMP at a familiar location with an endorsement of program benefit from trusted sources suggests the importance of relationship and expectations. Findings shed light on factors that may cause aging African Americans to embrace or avoid CDSMP as a healthcare option.

